# Identity of *bla*_CTX-M_ Carrying Plasmids in Sequential ESBL-*E. coli* Isolates from Patients with Recurrent Urinary Tract Infections

**DOI:** 10.3390/microorganisms9061138

**Published:** 2021-05-25

**Authors:** Nahid Karami, Sriram KK, Shora Yazdanshenas, Yii-Lih Lin, Daniel Jaén-Luchoro, Elina Ekedahl, Sanjana Parameshwaran, Anna Lindblom, Christina Åhrén, Fredrik Westerlund

**Affiliations:** 1 Institute of Biomedicine, Department of Infectious Diseases and Centre for Antibiotic Resistance Research (CARe), University of Gothenburg, Guldhedsgatan 10 A, 413 46 Gothenburg, Sweden; shora.yazdanshenas@vgregion.se (S.Y.); daniel.jaen.luchoro@gu.se (D.J.-L.); anna.u.lindblom@vgregion.se (A.L.); christina.ahren@vgregion.se (C.Å.); 2 Västra Götaland Region, Sahlgrenska University Hospital, Department of Clinical Microbiology, Guldhedsgatan 10A, 413 46 Gothenburg, Sweden; 3 Department of Biology and Biological Engineering, Chalmers University of Technology, Kemivagen 10, 412 96 Gothenburg, Sweden; sriramk@chalmers.se (S.K.); yiilih@gmail.com (Y.-L.L.); elina.ekedahl@hotmail.com (E.E.); sanjanaparameshwaran@gmail.com (S.P.); 4 Swedish Strategic Program against Antimicrobial Resistance (Strama), Västra Götaland Region, Regionens Hus, 405 44 Gothenburg, Sweden

**Keywords:** extended-spectrum beta-lactamase (ESBL), recurrent urinary tract infection, plasmids, horizontal transfer, *E. coli*, optical DNA mapping (ODM)

## Abstract

Plasmid-mediated multidrug resistance in *E. coli* is becoming increasingly prevalent. Considering this global threat to human health, it is important to understand how plasmid-mediated resistance spreads. From a cohort of 123 patients with recurrent urinary tract infections (RUTI) due to extended spectrum beta-lactamase (ESBL)-producing *Escherichia coli* (ESBL *E. coli*), only five events with a change of ESBL *E. coli* strain between RUTI episodes were identified. Their *bla*_CTX-M_ encoding plasmids were compared within each pair of isolates using optical DNA mapping (ODM) and PCR-based replicon typing. Despite similar *bla*_CTX-M_ genes and replicon types, ODM detected only one case with identical plasmids in the sequential ESBL *E. coli* strains, indicating that plasmid transfer could have occurred. For comparison, plasmids from seven patients with the same ESBL *E. coli* strain reoccurring in both episodes were analyzed. These plasmids (encoding *bla*_CTX-M-3_, *bla*_CTX-M-14_, and *bla*_CTX-M-15_) were unaltered for up to six months between recurrent infections. Thus, transmission of *bla*_CTX-M_ plasmids appears to be a rare event during the course of RUTI. Despite the limited number (*n* = 23) of plasmids investigated, similar *bla*_CTX-M_-_15_ plasmids in unrelated isolates from different patients were detected, suggesting that some successful plasmids could be associated with specific strains, or are more easily transmitted.

## 1. Introduction

Infections with extended-spectrum beta-lactamase (ESBL)-producing bacteria represent a serious challenge for modern healthcare systems, and are associated with high mortality rates and healthcare-related costs [1]. The most prevalent ESBL-producing bacteria are, by far, ESBL-producing *Escherichia coli* (ESBL *E. coli*), mostly causing urinary tract infections (UTI), but also more severe extraintestinal infections, such as septicemia [2]. We have found that almost one third of the patients with UTI due to ESBL *E. coli*, develop recurrent ESBL *E. coli* UTI (RUTI) [3]. Of particular concern is the highly virulent ESBL *E. coli* lineage of sequence type (ST) 131 that has reached pandemic proportions worldwide [4]. We recently reported its dominance in recurrent UTI (RUTI) caused by ESBL *E. coli* [5].

Most ESBL *E. coli* carry ESBLs of the CTX-M beta-lactamase types [2,6]. The *bla*_CTX-M_ genes are generally encoded on easily transferable plasmids and often carry resistance genes to other classes of antibiotics [6], resulting in immediate multidrug resistance if the plasmid is horizontally spread. The *bla*_CTX-M_ group 1 and 9 enzymes, including *bla*_CTX-M-15_ and *bla*_CTX-M-27_, respectively, are globally the most common ESBLs [6,7]. They are generally carried by plasmids that belong to the IncF family [8]. The plasmids vary in size (typically 50–200 kb) and replicon type, the most common types being IncFIA, IncFIB, and IncFII, where IncFII is often found in association with IncFIA and IncFIB [9]. IncF family plasmids also commonly carry addiction systems that promote their stability and maintenance in the bacterial host [10,11].

Horizontal transfer of plasmids carrying antibiotic resistance genes contributes to the increase of antibiotic-resistant infections worldwide [2,8]. Transfer of these plasmids between coexisting bacteria, for instance in the gut, is known to occur in vivo, especially under antibiotic pressure. The role of interspecies plasmid spread of resistance genes in polyclonal outbreaks of ESBL- or carbapenemase-producing Enterobacterales is also well documented [12,13].

Traditional methods for plasmid characterization are increasingly replaced by next-generation DNA sequencing (NGS) approaches. NGS is, however, time consuming and still demands high knowledge of bioinformatics, especially for the complete assembly of plasmid sequences. Optical DNA mapping (ODM) offers a rapid and simple way to analyze and compare plasmids [12,14]. The methodology relies on one-step labeling of DNA molecules using the intercalator dye YOYO-1 and netropsin that binds preferably to AT-rich regions. This results in an inhomogeneous emission intensity along the DNA that reflects the underlying sequence [14,15]. When stretching the DNA in nanofluidic channels, this variation in intensity, that we here refer as a “DNA barcode”, can be recorded [16]. In combination with CRISPR/Cas9 restriction, we can obtain information about plasmid size, resistance gene location, and barcode in the same experiment [17]. For the characterization of plasmids, we have previously demonstrated that the ODM results correlate very well with NGS analysis [14,18,19,20]. We have also showed its usefulness in tracking possible transmission routes in nosocomial outbreak investigations, in a neonatal polyclonal outbreak due to CTX-M-producing bacteria in Sweden, and in an ESBL *E. coli* ST410 outbreak in a clinical setting in Ethiopia [12,20].

The dynamics of the *bla*_CTX-M_ carrying plasmids during the course of recurrent infections with ESBL *E. coli* are, to our knowledge, largely unexplored. To fully understand the entire pathophysiology of RUTI due to ESBL *E. coli*, the likelihood of a possible horizontal transfer of the *bla*_CTX-M_ encoding plasmid between the bacteria causing the first and the following RUTI episodes needs to be explored. We recently reported that plasmid transmission appears to be a rare event when the sequential isolates in RUTI are of different species, that is *E. coli* and *Klebsiella pneumoniae*, though we could demonstrate a few such possible events using ODM [21].

In this study, we analyzed pairs of *bla*_CTX-M_ carrying plasmids from sequential ESBL *E. coli* isolates from patients with RUTI using ODM. Isolates from patients with either a change or no change in *E. coli* strain between RUTI episodes were included. The aim was to explore the likelihood of within-species horizontal *bla*_CTX-M_ carrying plasmid transfer during the course of RUTI. Firstly, plasmids from patients with a change of ESBL *E. coli* strain type between RUTI episodes were compared. Secondly, plasmids from patients with the same ESBL *E. coli* strain reoccurring at each episode were analyzed as a reference group to the first group of patients. In all these cases, only patients with isolates of an identical *bla*_CTX-M_ gene in the first and subsequent RUTI episode were included, as this was taken as an indication that *bla*_CTX-M_ carrying plasmids transfer possibly could have occurred.

In concordance with our previous study [21], ODM analysis demonstrated that transmission of *bla*_CTX-M_ carrying plasmids seems to be a rare event during the course of RUTI caused by CTX-M-producing bacteria in a patient. We also found all *bla*_CTX-M_ carrying plasmids to be stable over time when the same ESBL *E. coli* strain reoccurred in the following episode, which it does in a majority of patients with RUTI. Interestingly, subsets of unrelated isolates with similar *bla*_CTX-M-15_ carrying plasmids were identified. This could indicate that some successful *bla*_CTX-M-15_ carrying plasmids could be associated with specific strains, or are more easily transmitted.

## 2. Materials and Methods

### 2.1. Patients and Isolates

The 23 isolates in this study were part of a previous study investigating the bacterial features in 356 ESBL *E. coli* urinary isolates obtained for diagnostic purposes at the Clinical Microbiology Laboratory, Sahlgrenska University Hospital, Gothenburg, Sweden between 2004 and 2014 [5]. The isolates were obtained from 123 patients with RUTI within a year (2–8 episodes/patient). Ten isolates represented all detected events (*n* = 5) in the entire study population, where a change in ESBL *E. coli* strain type had occurred between the RUTI episodes. The remaining isolates, serving as a reference group, represented events where the same strain reoccurred in all episodes. Here, we aimed for diversity in STs, phylogroup, and *bla*_CTX-M_ gene for the included isolates. The timespan was at least 30 days between each RUTI episode. No additional urinary cultures positive for any uropathogen in between the studied episodes was noted. Information of the large patient cohort and bacterial typing results are detailed in a previous report [5]. The laboratory covered all healthcare units, both in the outpatient and hospital settings in the greater Gothenburg region. Epidemiological and clinical data linked to the isolates are documented in the laboratory database. No epidemiological relationship between the patients in this study was present.

The *E. coli* isolates were identified according to routine clinical microbiology practice. The disk diffusion method was used for antimicrobial susceptibility testing according to the recommendation of the European Committee on Antimicrobial Susceptibility Testing (EUCAST) at the time [22]. Cephalosporin-resistant isolates were screened for ESBL phenotype by the double-disk diffusion assay and ESBL-positive isolates were stored at −70 °C. Frozen isolates were retrieved, plated on blood agar medium, and incubated overnight at 37 °C. From the bacterial culture, DNA was extracted as previously described, and was used for subsequent DNA analyses [23].

### 2.2. Phylogenetics Analysis of ESBL E. coli

The phylogroup of each *E. coli* isolatess was determined using the updated Clermont method presented in 2013 [24]. PFGE typing was performed as described previously [25]. Strains with fewer than four band differences and ≥80% similarity index were considered as the same strain type according to the criteria of Tenover et al. [26]. Multilocus sequence typing (MLST) was performed according to the method of the MLST database website for ESBL *E. coli* [27].

### 2.3. Detection of bla_CTX-M_ Genes

All isolates were investigated first for *bla*_CTX-M_ group, i.e., group 1, 2, and 9, using a TaqMan PCR protocol [28]. Thereafter, sequencing was performed to determine the presence of specific *bla*_CTX-M_ genes in the studied isolates, and in the subsequent plasmid preparation.

### 2.4. Plasmid Extraction and Replicon Typing of Plasmids

Plasmid DNA was prepared from overnight culture with the NucleoBond Xtra Midi Kit (Macherey–Nagel, Duren, Germany) according to the manufacturer’s instructions for low-copy plasmids. The plasmid DNA was precipitated with isopropanol and washed once with 70% ethanol. The dried pellet was reconstituted in 50 μL TE-buffer. DNA concentration and purity were determined using NanoDrop and Qubit™ dsDNA BR Assay Kit (Thermo Fisher, Waltham, MA, USA). Incompatibility typing of the plasmids to determine replicon types was performed by PCR [29].

### 2.5. Optical DNA Mapping of Plasmids

The ODM methodology for analyzing plasmids has been demonstrated previously [12,21]. In brief, plasmid DNA isolates were subjected to CRISPR/Cas9 mediated restriction in the presence of gRNA, targeting either *bla*_CTX-M_ group 9 (5′AGAGAGCCGCCGCGATGTGC3′) or *bla*_CTX-M_ group 1 (5′CCGTCGCGATGTATTAGCGT3′) [17]. gRNA was obtained by mixing equimolar amounts of crRNA and tracrRNA (0.5 nmol each, Dharmacon Inc., Lafayette, CO, USA) in the presence of 1X NEB-3 buffer (New England Biolabs, Ipswich, MA, USA) and 1× bovine serum albumin (BSA, 0.1 µg/mL), and incubating at 4 °C for 30 min. To this mixture, Cas9 protein (600 ng, Sigma Aldrich, St. Louis, MI, USA) was added and incubated at 37 °C for 15 min to form Cas9–gRNA complexes. Further, plasmid DNA (60 ng) was added to the tube containing the Cas9–gRNA mixture and allowed to incubate at 37 °C for 1 h to allow the Cas9–gRNA complex to restrict the plasmid DNA molecules at the gene site [17].

Next, samples were mixed with YOYO-1 (1:2 bp molar ratio, Invitrogen) and netropsin (60:1 bp molar ratio, Sigma-Aldrich) to achieve one-step fluorescence labelling. λ-DNA (48,502 bp, New England Biolabs) was used as an internal size reference [30]. In this step, 0.5× Tris-borate-EDTA (TBE, Sigma-Aldrich) buffer was used as the reaction buffer, and the mixture was incubated at 50 °C for 30 min to achieve homogenous labelling. The sample was further diluted with MilliQ water in the presence of 3% (*v*/*v*) of β-mercaptoethanol (BME, Sigma-Aldrich), to ensure the working sample was in 0.05× TBE for effective stretching of DNA in nanochannels, with BME being responsible for minimizing photodamage of DNA while recording the images.

The nanofluidic devices and the fabrication procedure are discussed in detail elsewhere [31]. Briefly, the device consisted of four loading reservoirs, with one microchannel connecting the top two reservoirs and another microchannel connecting the bottom two reservoirs. A total of 200 nanochannels of 150 nm width, 100 nm depth, and 500 µm length, connecting the two microchannels, enabled the stretching of the DNA molecules due to the nanoconfinement, forming the basis of the ODM methodology. The chip was connected to a chuck, custom-made to fit on top of an epifluorescence microscope (Zeiss AxioObserver.Z1), and images were collected using a 100× oil immersion objective (Zeiss, NA = 1.46), a FITC filter (488 nm excitation/530 nm emission), and an sCMOS camera (Photometrix Prime 95B). The chuck was designed with one pressure inlet to each of the four reservoirs, enabling pressure-driven flow of the DNA molecules.

Experiments were performed by loading a sample in one of the reservoirs, with the other three reservoirs being filled with 0.05× TBE containing 3% (*v*/*v*) BME. Positive pressure was applied to drive the solution containing DNA molecules first into the microchannel, followed by a pressure applied across the microchannels to drive the DNA molecules into the nanochannels. When the DNA had entered the nanochannels, the pressure was switched off and images were recorded of relaxed molecules. Images were obtained for 20 frames, with an exposure time of 100 ms. Collected images were saved in TIFF file format and further analyzed with help of custom developed MATLAB codes.

The analysis procedure used here is explained in detail elsewhere [32]. Briefly, we extracted the emission intensity variations, the DNA barcodes, along the single DNA molecules from the TIFF files. The intensity variation traces were compared and clustered based on their similarity, and a consensus trace was generated from the cluster. Only traces with an identical site of Cas9 restriction, confirming the presence of the resistance gene of interest in that plasmid, were used [17]. Plasmid lengths were measured by using λ-DNA of known length as reference. Subsequently, an experiment-to-experiment comparison was carried out between isolates of the same patient, as well as across different patients, to investigate if the plasmids were identical. For all comparisons between plasmids, the lengths obtained from the ODM experiments were used. A *p*-value of 0.01 or lower was used as a cut-off to determine that two plasmids have a “good match”. Plasmids that were of the same length and barcodes, with genes located at the same location (within the resolution of ODM technique, which can be few kb) were considered identical plasmids. Partially identical plasmids were ones with identical barcodes and gene locations, but different lengths due to insertions or deletions. Plasmids carrying the genes at different locations (even if barcodes are similar) were considered non-identical.

## 3. Results

Altogether 23 isolates from 11 patients with recurrent ESBL *E. coli* UTI were chosen from our previous study [5], describing bacterial strain characteristics in 356 ESBL *E. coli* isolates from 123 patients with at least two episodes of ESBL *E. coli* UTI within a year (Table 1).

In this large cohort, we could identify altogether five patients where a change of ESBL *E. coli* strain between two consecutive RUTI episodes had occurred. The change in strain type was confirmed by a change in pulsed-field gel electrophoresis (PFGE) type in all cases and, in four of them, the *E. coli* sequence type was also different (Table 1). The *bla*_CTX-M_ genes in the sequential UTI isolates were, however, identical, indicating that horizontal transfer of *bla*_CTX-M_ plasmids possibly could have occurred prior to the second RUTI episode. In addition, the subsequent isolates all carried plasmids of the same replicon types as their previous counterpart.

**Figure 1:**
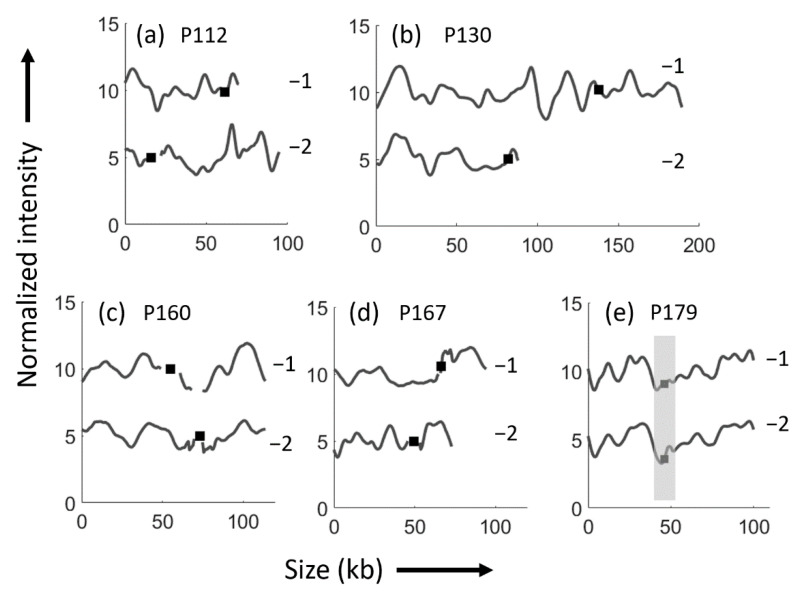
ODM results of ESBL *E. coli* isolates analyzed for *bla*_CTX-M_ gene in patients with recurrent urinary tract infections caused by different ESBL *E. coli* strains. (**a**) Patient P112 with nonidentical *bla*_CTX-M-27_ encoding plasmids in P112-1 (70 kb), and P112-2 (86 kb). (**b**) Patient P130 with nonidentical *bla*_CTX-M-15_ encoding plasmids in P130-1 (189 kb) and P130-2 (93 kb). (**c**) Patient P160 with nonidentical *bla*_CTX-M-27_ encoding plasmids in P160-1 (104 kb) and P160-2 (114 kb). (**d**) Patient P167 with nonidentical *bla*_CTX-M-27_ encoding plasmids in P167-1 (89 kb) and P167-2 (73 kb). (**e**) Patient P179 with identical *bla*_CTX-M-27_ encoding plasmids in P179-1 and P179-2 (100 kb). Black squares indicate the location of the gene and the region shaded in grey indicates that the gene is in the same location in figure e.

To investigate the likelihood of potential plasmid transfer between isolates, we turned to ODM analysis. For four of the patients with a new ESBL *E. coli* strain in the following episode, the plasmid barcodes and the *bla*_CTX-M_ gene locations did not match (Figure 1). In one case (P130, the two first episodes in the Table 1), there was a good match between the barcodes, but the lengths were very different and the *bla*_CTX-M_ gene locations varied, suggesting that the two plasmids were not identical. Only for one patient (P179), we observed identical plasmids with the same size and barcode, and an identical placement of the *bla*_CTX-M-15_ gene along the plasmids in the two consecutive strains, indicating that plasmid transmission possibly could have occurred during the course of RUTI.

For comparison, we also analyzed isolates from seven patients where the same strain, according to PFGE analysis, reoccurred between the sequential episodes and with the same *bla*_CTX-M_ gene in the two pairwise compared isolates (Table 1). We aimed to include isolates of various phylogroups, STs, and *bla*_CTX-M_ genes, but no patient with a reoccurring *bla*_CTX-M-27_ carrying strain could be identified from the large cohort of patients with RUTI.

**Figure 2:**
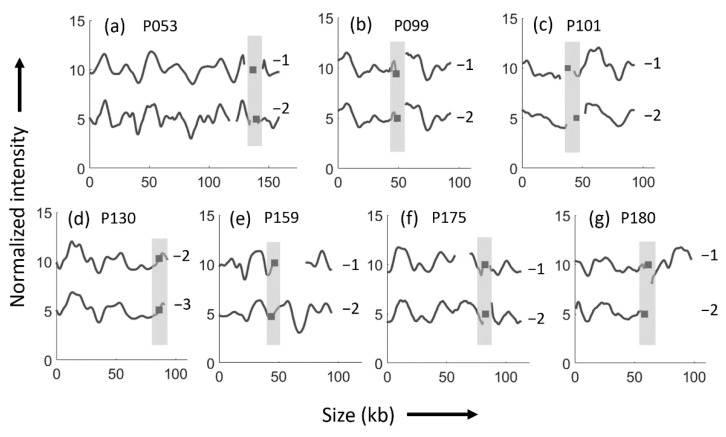
ODM results of ESBL *E. coli* isolates analyzed for bla_CTX-M_ gene in seven patients with consecutive episodes of recurrent urinary tract infections caused by the same ESBL *E. coli* strains. ODM barcodes for (**a**) patient P053, with ~150 kb plasmid carrying the *bla*_CTX-M-15_ gene at the same location in both isolates; (**b**) patient P099, with identical 93 kb plasmids carrying the *bla*_CTX-M-14_ gene at the same location in both isolates; (**c**) patient P101, with ~90 kb plasmids carrying the *bla*_CTX-M-15_ gene at the same location in both isolates; (**d**) patient P130 (isolates P130-2 and P130-3), with identical plasmids of 93 kb length carrying the *bla*_CTX-M-15_ at the same location in both isolates; (**e**) patient P159, with plasmids of different size (P159-1; 71 kb and P159-2; 95 kb) carrying *bla*_CTX-M-3_ gene at the same location in both isolates; (**f**) patient P175, with plasmids of different size (P175-1; 104 kb and P175-2; 113 kb) carrying the bla_CTX-M-14_ gene at the same location in both isolates; (**g**) patient P180, with plasmids of different size (P180-1; 98 kb and P180-2; 62 kb) carrying the bla_CTX-M-15_ gene at the same location in both isolates. Black squares indicate the location of the gene and the regions shaded in grey indicates that the gene is in the same location.

The isolates from the patients with the same ESBL *E. coli* strain reoccurring in each episode were all found to carry plasmids of the same replicon type, matching ODM barcode, and *bla*_CTX-M_ gene position. The length of the plasmids differed to some extent in all but two pairs of isolates (P099 and P130), suggesting that insertions or deletions of varying sizes had occurred in the plasmids between the two RUTI episodes (Figure 2).

Patient 130 (Table 1) was included in both Figure 1 and Figure 2 since that patient had three RUTI episodes, first with different strains (episode one and two), and thereafter with the same strain (episode two and three). The plasmids differed between the first and second strain (Figure 1b), but were identical at the last two episodes caused by the same strain (Figure 2d).

**Figure 3:**
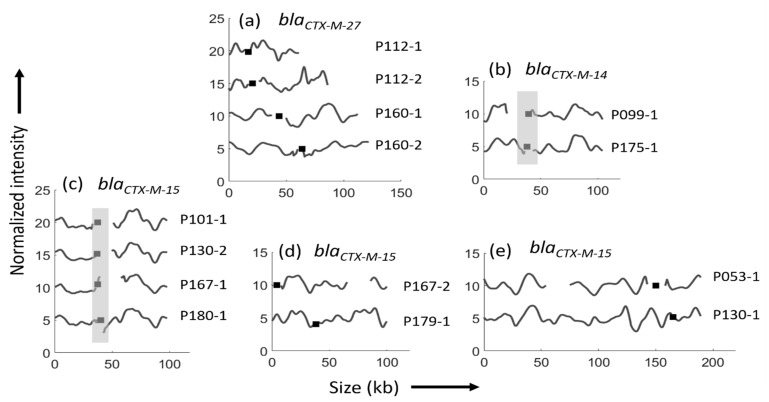
ODM barcodes and bla_CTX-M_ gene location of plasmids from different isolates and patients grouped by the specific *bla*_CTX-M_ gene. (**a**) Four completely different plasmids encoding the *bla*_CTX-M-27_ gene. (**b**) Similar plasmids of different length encoding the *bla*_CTX-M-14_ gene. (**c**) Highly similar plasmids, approximately 90 kb in length, encoding the *bla*_CTX-M-15_ with gene at same gene location, obtained from four different isolates and patients. (**d**) Plasmids encoding the *bla*_CTX-M-15_ gene from two isolates with similarity in barcodes, but with different gene locations and different lengths (73 kb and 100 kb, respectively). (**e**) Plasmids encoding the *bla*_CTX-M-15_ gene from two isolates showing partial similarity in barcodes, but with different gene locations and different lengths (159 kb and 189 kb, respectively). Black squares indicate the location of the gene and the regions shaded in grey indicates that the gene is in the same location.

In a final step, we compared plasmids with the same *bla*_CTX-M_ genes across the patients (Figure 3). In case of identical strains and plasmids in the sequential isolates from a patient, only the first of the two isolates were included.

For *bla*_CTX-M-27_, all four plasmids from two patients were different, and all came from isolates of different STs, not even of the same clonal complexes (Figure 3a, Table 1). The plasmids encoding *bla*_CTX-M-14_ (P099-1 and P175-1) had similar ODM barcodes (*p* < 0.01) with the *bla*_CTX-M-14_ gene at the same location, but the latter (P175-1) was 22 kb longer, thus, they were partially identical (Figure 3b, Table 1). These similarities could indicate a previous common origin.

A comparison between the eight plasmids encoding *bla*_CTX-M-15_ (representing five STs and three phylogroups) yielded three different subsets of plasmids. The first subset (P101-1, P130-2, P167-1, and P180-1) showed high similarities (*p* < 0.01), with similar size (~90 kb) and *bla*_CTX-M-15_ gene location (Figure 3c, Table 1). Within the other two subsets of plasmids, a high degree of similarity for the ODM barcode was found, but the plasmids were nonidentical as the length varied and the *bla*_CTX-M-15_ gene position was different in the two pairs. P167-2 and P179-1 were nonidentical, with similar ODM barcodes, but a difference in size by ~27 kb and the *bla*_CTX-M-15_ gene at different locations (Figure 3d). The long plasmids in P053-1 and P130-1 had similar barcodes, but the gene position and the size (P130-1 is ~30 kb longer than P053-1) were different (Figure 3e). For the latter two comparisons, the fact that the barcodes were similar but the gene position and size different, suggests that the plasmid could have a common origin, but this is likely not a recent event considering the significant differences.

## 4. Discussion

The goal of this study was to analyze plasmids from patients with RUTI to investigate if plasmids, with identical *bla*_CTX-M_ genes identified in consecutive ESBL *E. coli* isolates of different strain types, were the same from one to the next RUTI episode in a patient. If so, this could indicate that the second ESBL *E. coli* strain had obtained its *bla*_CTX-M_ plasmid from the first ESBL *E. coli* strain via horizontal plasmid transfer prior to causing the second RUTI episode, for instance, in the patient gut flora. From a large cohort of 123 patients with two or more UTI episodes due to ESBL *E. coli*, only five such possible events could be identified, and only in one case did the sequential strains share an identical *bla*_CTX-M-15_ encoding plasmid. Thus, horizontal *bla*_CTX-M_ plasmid transfer between sequential RUTI strains in a patient appears to be a rare event. This is in accordance with our previous findings, where *bla*_CTX-M_ plasmid transfer between sequential CTX-M-producing bacteria of different species appeared to be very rare in patients with RUTI [21]. Both studies were carried out in an ESBL *E. coli* low endemic setting, with a low risk of constant reinfections with new CTX-M-producing bacteria, indeed a prerequisite for this type of study. Nonetheless, the most likely explanation for the few cases with an observed change of strain between RUTI episodes is that the patient has been infected with a new strain already carrying a *bla*_CTX-M_ plasmid, in this study, a new ESBL *E. coli* strain subsequently causing disease.

We cannot exclude that, in the one case (P179) with identical *bla*_CTX-M-15_ plasmids despite a change of ESBL *E. coli* strain between episodes, that the two strains had acquired their plasmids as separate events prior to infecting the patient, and that no transmission of *bla*_CTX-M_ plasmid had occurred during the course of RUTI. Considering the polyclonal nature of ESBL *E. coli*, with a large variation of ESBL *E. coli* STs in our region at the time, and the clear dominance of a few CTX-M enzymes, in particular CTX-M-15, this could well be the case and, thus, further strengthens our conclusion [25]. We also found an isolate from another patient (P167-2) with a nonidentical *bla*_CTX-M-15_ plasmid, but with a similar ODM barcode to those of P179, indicating a possible common source of plasmid, but not of recent origin. P167-2 was an ST131 isolate, which is the most prevalent ST in ESBL *E. coli* [33], meaning that this plasmid could have been readily available and obtained at a previous occasion.

We complemented the study with isolates from patients where the consecutive RUTI episodes were caused by the same bacterial strain. In this sample set, including isolates of different phylogroups and both rare and common STs in ESBL *E. coli* [33], we observed that identical plasmids were present in the consecutive isolates in all seven cases, including isolates with *bla*_CTX-M-3_, *bla*_CTX-M-14_, and *bla*_CTX-M-15_, respectively. Interestingly, in some cases, minor differences were observed in size between these pairwise plasmids carried by the same strain type. This was readily detected thanks to the ODM technique and can be attributed to deletions or insertions between the RUTI episodes. The stability of the plasmids is well in line with the discussed stability of IncF-plasmids by Bahl et al. [10] and studies describing high *bla*_CTX-M_ plasmid stability in isolates of the ST131 lineage [34,35]. It is also in accordance with our previous findings investigating longstanding outbreaks with ESBL *E. coli* using ODM [12,18].

This study also demonstrates the need for more advanced methods, like ODM, for detailed analysis of plasmids. Despite the same *bla*_CTX-M_ gene, replicon type, and similar size, the plasmids were not identical by ODM analysis in four of the studied pairs of ESBL *E. coli* strains of different strain types. This was detectable thanks to the ODM method that gives a detailed fingerprint for each plasmid that can be compared between isolates. A further advantage of the ODM method is the ability to overcome the plasticity of plasmids when analyzing plasmid identity over time, with insertions and deletions, as demonstrated also in our previous studies [18,20,21].

When analyzing all *bla*_CTX-M-15_ plasmids identified in this study, high similarities were identified within a subset of four isolates from different patients. Interestingly, three of these isolates belonged to common ESBL *E. coli* STs (ST131 and ST12), and three belonged to phylogroup B2, the most prevalent phylogroup in uropathogenic ESBL *E. coli* [33,36]. We also found similarities for the *bla*_CTX-M-14_ plasmids detected; again, one of these isolates belonged to ST131 and another common ST (ST69). A close clonal relationship within ST131 and specific plasmids has been reported previously [34,35], suggesting a strong coevolution of strains and plasmids within this conserved lineage, but little is known for other lineages.

Considering the design of the study, the number of isolates and plasmids was too limited to draw any conclusions of the diversity of *bla*_CTX-M_ plasmids in ESBL *E. coli* in general and how they may spread. Our aim in the present study was to investigate possible ESBL-plasmid transfer in cases with recurrent ESBL *E. coli* infections, and not the dissemination of *bla*_CTX-M_ encoding plasmids per se. The most likely place of horizontal ESBL-plasmid transfer in a patient is within the gut flora, and such transfer may very well occur without the result of a new virulent ESBL *E. coli* strain causing infection [37,38]. Considering the global increase of antimicrobial resistance, particularly in ESBL *E. coli*, it is important to further elucidate the diversity of the *bla*_CTX-M_ plasmids and to which extent clones and plasmids coevolve, not only for the ST131 lineage, but also for other important ESBL *E. coli* lineages that cause disease. Considering the constant emergence of new *bla*_CTX-M_ genes and clones, such as the more recently described *bla*_CTX-M-27_ in ST131 [7], it will also be important to evaluate a change over time locally, as well as globally. In this respect, the ODM technique offers a simple tool to analyze plasmids in a large number of isolates.

The present study has limitations. The isolates tested are part of a laboratory-based, retrospectively identified cohort of patients. The available isolates have been stored in an arbitrary way, and isolates from patients with RUTI in our database are missing, so there could have been additional undetected episodes with a change in strain type. Moreover, the number of included isolates from patients with repeated infections with the same strain were limited and were not selected to represent the entire cohort of patients with RUTI, as this would have meant including similar strains, especially those of phylotype B2 and ST131. Instead, we aimed for diversity of strain and *bla*_CTX-M_ types.

To conclude, from our studies of patients with RUTI caused by CTX-M-producing bacteria, a change of *bla*_CTX-M_ encoding plasmid appears to be a very rare event during the course of RUTI, at least for the first six months when most recurrences occur [3]. Instead, the bacterial strain and its *bla*_CTX-M_ encoding plasmid seems surprisingly stable over time. The value of ESBL-plasmid characterization and keeping track of these plasmids, in addition to strain typing and resistance determination, to guide clinicians in evaluating recurrent ESBL *E. coli* infections in the general patient, thus, appears low.

## Figures and Tables

**Table 1:** Detailed characteristics of 23 ESBL *E. coli* isolates analyzed for *bla*_CTX-M_ plasmid identity from 11 patients with recurrent urinary tract infection caused by the same or different ESBL *E. coli* strains in the consecutive UTI episodes.

Patient and Isolate	Year of Isolation	Sex/Age (Year)	Time from the First Isolate (Month)	Consecutive Isolate Identical ^1^	ST	PFGE—Similarity (%) ^2^	Phylo Group	CTX-M Gene	Replicon Type (Inc)	Size of Plasmid (kb)
P112-1	2008	M/65	-		131	50	B2	27	FIA, FIB, FII, N	70
-2			2	-	141		B2	27	FII, N	86
P160-1	2011	F/34	-		58	50	B2	27	FIA, FIB, FII, I1, Y, B/O	104
-2			4	-	43		B1	27	FIB, FII, B/O	114
P167-1	2009	F/20	-		2141	45	F	15	FIA, FIB, FII, I1	89
-2			1	-	131		B2	15	FIA, FIB, FII	73
P179-1	2011	F/2	-		394	44	D	15	FII, K/B	100
-2			6	-	80		B2	15	K/B	100
P130-1	2009	F/59	-		131	61	B2	15	FIA, FIB, FII, B/O	189
-2			6	-	131		B2	15	FIA, FIB, FII, I1	93
-3 ^3^			8	+		100	B2	15	FIA, FIB, FII, I1	93
P053-1	2013	F/85	-		617	100	A	15	FIA, FIB, FII	150
-2			5	+			A	15	FIA, FIB, FII	150
P099-1	2010	F/71	-		69	83	D	14	FIB, FII, B/O	93
-2			6	+			D	14	FIB, FII	93
P101-1	2010	F/71	-		12	90	B2	15	FII, I1	94
-2			5	+			B2	15	FII, I1	82
P159-1	2010	M/33	-		354	90	F	3	FIA, FIB, FII	71
-2			6	+			F	3	FIA, FIB, FII	95
P175-1	2009	M/2	-		131	97	B2	14	FIA, FIB, FII I1	104
-2			5	+			B2	14	FIA, FIB, FII I1	113
P180-1	2012	M/1	-		131	95	B2	15	FIA, FIB, FII	98
-2			5	+			B2	15	FIA, FII	62

^1^ When a different strain was detected this is indicated by -, and if the same strain was detected in the consecutive episodes in a patient, this is indicated by + for the isolate at the following episode. ^2^ PFGE pattern similarity between index and subsequent isolates. The cut-off point for different PFGE types was 80% similarity. ^3^ A third RUTI episode with an isolate identical (100%) to the second isolate was also detected 2 months later in this patient.

## Data Availability

The ODM data can be obtained from the corresponding authors upon reasonable request.
